# Effects of chili straw on rumen fermentation, meat quality, amino acid and fatty acid contents, and rumen bacteria diversity in sheep

**DOI:** 10.3389/fmicb.2024.1525612

**Published:** 2025-01-14

**Authors:** Jinlong Li, Yong Tuo, Linjiao He, Yan Ma, Zhijun Zhang, Zhiqiang Cheng, Changjiang Zang, Tongjun Guo

**Affiliations:** ^1^Feed Research Institute of Xinjiang Academy of Animal Husbandry Sciences, Urumqi, China; ^2^College of Animal Science, Xinjiang Agricultural University, Urumqi, China; ^3^Xinjiang Key Laboratory of Herbivorous Livestock Feed Biotechnology, Urumqi, China

**Keywords:** chili straw, Dorper×Hu hybrid sheep, rumen bacteria, meat quality, fatty acids

## Abstract

Crop residues have shown promise as non-conventional feed sources to enhance animal health and growth. This study evaluated the effects of chili straw (CS) on rumen fermentation, meat quality, amino and fatty acid composition, and rumen microbial diversity in sheep. Fifty F1 Dorper×Hu lambs (29.58 ± 2.06 kg) were randomly assigned to five groups, fed pelleted feed with 0%, 5%, 10%, 15%, or 20% CS over a 63-day period, including a 7-day pre-test. Post-trial, rumen fluid was sampled to assess fermentation and microbial profiles, and slaughter performance and meat quality were evaluated. Key findings include: (1) No significant differences were observed in rumen pH, NH_3_-N, or acetic acid-to-propionic acid ratio across groups (*P* > 0.05). (2) Rumen microbial diversity indices did not vary significantly between groups (*P* > 0.05), though the relative abundance of Firmicutes and Proteobacteria increased, and *Bacteroidota* decreased in CS-fed groups, with specific genus-level changes. (3) Carcass weight decreased in the CS20% group (*P* < 0.01). (4) Cooking loss decreased in CS10%, 15%, and 20% groups (*P* < 0.05), and meat redness increased in CS15% and 20% groups (*P* < 0.01). (5) Saturated fatty acids decreased, while the PUFA/SFA ratio and amino acid profiles, including sulfur-containing amino acids (SAA), dibasic amino acids (DAA), essential amino acids (EAA), and total amino acids (TAA), increased with CS, with a significant rise in Gly content in the CS15% group (*P* < 0.05). In conclusion, incorporating CS into lamb diets can enhance meat quality without adversely affecting rumen fermentation, with recommended levels between 10% and 15%.

## 1 Introduction

China's animal husbandry is currently experiencing rapid development, with feed accounting for a large proportion of the total production cost. Ruminant breeding requires large amount of roughage, but China faces limitations in both quantity and quality of roughage resources, which has significantly hindered the progress of local livestock farming. High-quality roughage such as alfalfa and oats must be imported, while locally abundant unconventional roughage resources such as straw, leaves, and fruit peels are wasted through disposal methods like burning, which contributes to environmental pollution (Xing et al., 2017). Hence, the exploration of unconventional, safe, and sustainable feed resources is of great importance for advancing the livestock industry.

Chili is a perennial herbaceous plant of the Solanaceae family, known for its high yield. Based on data from the Food and Agriculture Organization of the United Nations, China's chili planting area was ~759,800 hectares, with an annual output of 16,833,740 tons, ranking first in the world. As a by-product of chili cultivation, China produces 7,246,800 tons of chili straw (CS) each year, which is a very rich but little utilized resource. The crude protein (CP), neutral detergent fiber (NDF), acid detergent fiber (ADF), and acid detergent lignin (ADL) content of CS (fresh whole chili straw, including roots, stems, and leaves) were 14.79%−16.72%, 35.22%−43.31%, 33.81%−39.61%, and 13.64%−18.61%, respectively (Lu et al., 2012), and its nutritional value is close to that of alfalfa and higher than that of corn straw and wheat straw (Lu, 2013). CS not only provides a variety of nutrients, but also has multiple biological functions, which have a positive impact on improving livestock and poultry production performance, meat quality and microbial diversity (Bampidis et al., 2020; Cho et al., 2020). Studies have shown that adding 10% CS to the diet of Rex rabbits can increase their daily weight gain and improve the apparent digestibility of nutrients (Cheng J. F. et al., 2023). In addition, Lu (2023) found that adding CS to mutton sheep diet could increase the relative abundance of Firmicutes and reduce the relative abundance of Proteobacteria in the rumen, thereby improving the microbial environment. CS contains various bioactive compounds, including flavonoids, capsaicin, carotene, polysaccharides and polyphenols (Bampidis et al., 2020; Cho et al., 2020). Among them, flavonoids account for the largest proportion and have a range of bioactive properties, such as antioxidant, anti-inflammatory and antibacterial effects (Panche et al., 2016). The main active compounds among flavonoids are quercetin and luteolin (Van Zonneveld et al., 2015). Rahim (2020) demonstrated that a 10% flavonoid extract from chili leaves exhibited potent antibacterial activity against *Staphylococcus aureus*. CS also contains capsaicin and polyphenolic compounds, which are essential for effectively reducing inflammation, enhancing gut health, and promoting animal growth and overall health. Studies have shown that adding 8% chili straw to piglet feed can increase the activity of glutathione peroxidase (GSH-Px), superoxide dismutase (SOD), and total antioxidant capacity in serum, while reducing malondialdehyde (MDA) content (Zhao, 2018).

Currently, there are increasing studies on the application of CS as an unconventional roughage in monogastric animals. Studies have found that CS significantly impacts the growth performance, digestive metabolism, and meat quality of monogastric animals. However, the specific mechanisms by which CS affects rumen fermentation, fatty acids, and meat quality in ruminants is still unclear. Therefore, this study aims to build a scientific foundation and provide practical reference for the application of CS in ruminant production by exploring the effects of different levels of CS in the diet on rumen fermentation, rumen microbial community, meat quality, and fatty acid composition of sheep.

## 2 Materials and methods

All procedures related to animal care and handling in this study were performed in compliance with the Guidelines for the Care and Use of Laboratory Animals in China and approved by the Animal Care Committee of Xinjiang Academy of Animal Sciences, China (Protocol License No.3 20230507, Urumqi, China).

### 2.1 Experimental materials

Chili straw is the air-dried roots and stems remaining after the fruit of Tianjiao Hongguan chili [registration number GPD chili (2017) 650019] is harvested. The chili straw used in this study was provided by Xinjiang Yuanda Green Agriculture Development Co., Ltd. (Kashgar, China). Its nutritional composition is detailed in Table 1.

**Table 1:** Nutritional composition of chili straw (%).

**Item**	**Nutritional content**
DM	92.02
CP	6.81
EE	1.13
NDF	54.66
ADF	38.17
Ash	7.16
Ca	1.01
P	0.12

Except for DM the content of other nutrients is based on dry matter.

**Figure 1:**
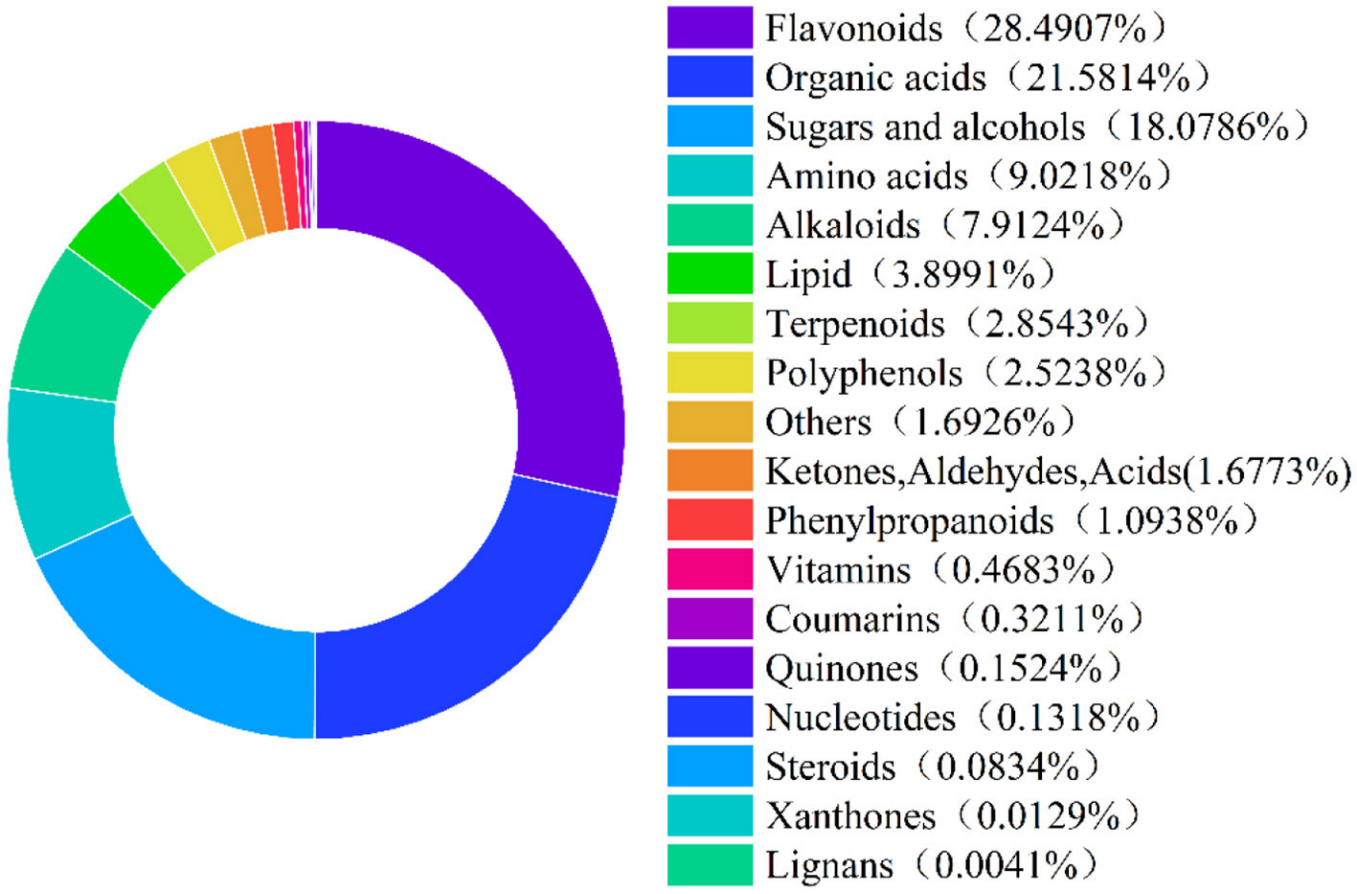
Classification of bioactive substances in chili straw. The percentages shown in the figure are the proportion of each bioactive compound to the total bioactive compound in the chili straw.

CS samples sent to Beijing BioMarker Biological Co., Ltd. for broad-spectrum plant metabolite analysis using liquid chromatography-mass spectrometry (LC-MS). Figure 1 shows the classification of bioactive substances in CS, with a total of 18 categories and 1,189 compounds were identified. Among them, flavonoids accounted for the largest proportion (28.49%), followed by organic acids (21.58%), sugars and alcohols (18.08%), amino acids (9.02%), and alkaloids (7.91%).

### 2.2 Experimental design, animal diets, and management

**Figure 2:**
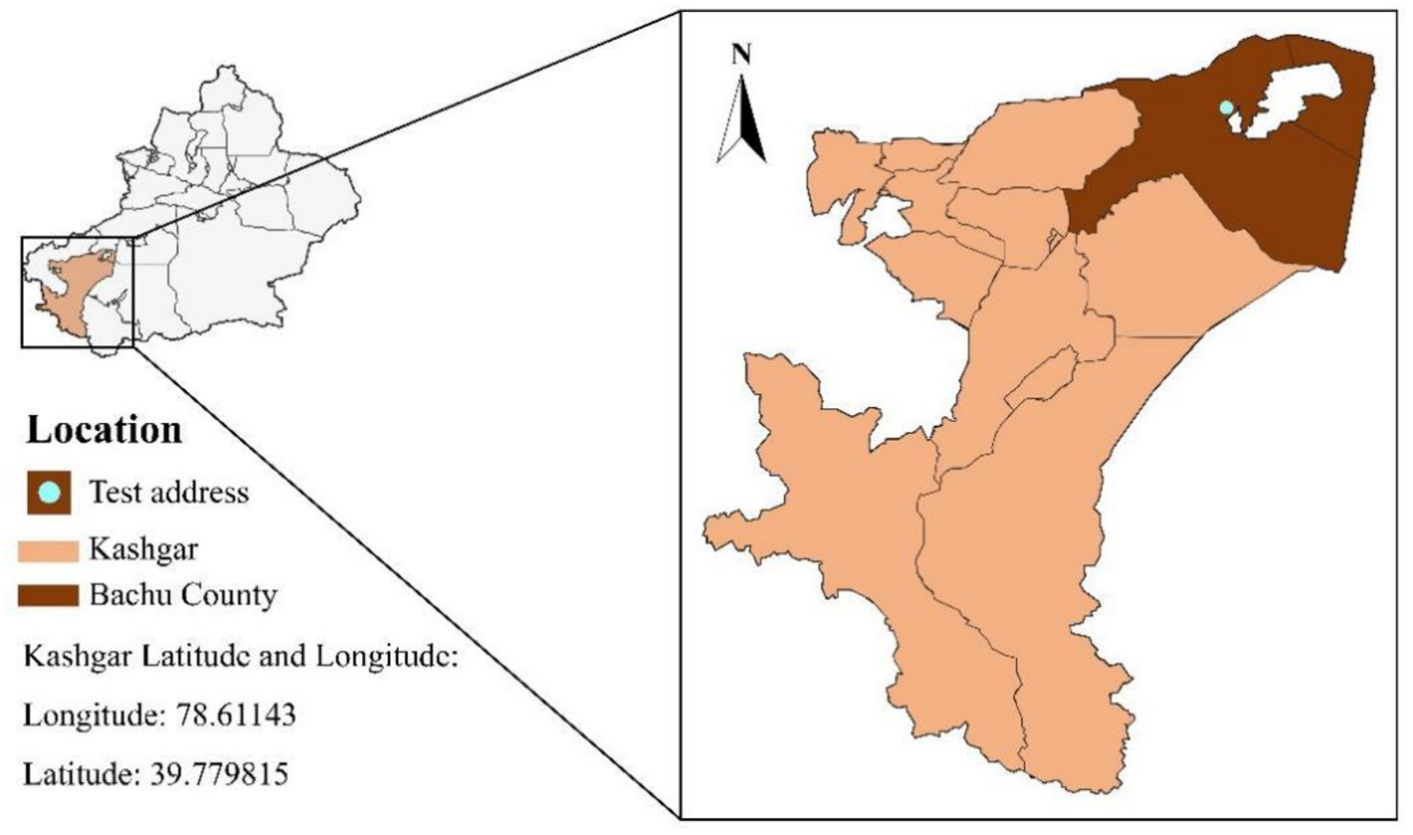
Test location at the Bachu County Taihe Agriculture and Animal Husbandry Technology Co., Ltd sheep farm in Xinjiang, China.

The animal experiments were conducted at the sheep farm of Xinjiang Taihe Agriculture and Animal Husbandry Technology Co., Ltd (Figure 2). In this experiment, 50 F1 hybrid lambs produced by the cross between Dorper sheep and Hu sheep were selected. They were 3–4 months old and had similar body weights (29.58 ± 2.06 kg). The lambs were randomly allocated into five groups: control (CON group), experimental group I (CS5%), experimental group II (CS10%), experimental group III (CS15%), and experimental group IV (CS20%), with ten lambs in each group. The control group was fed a standard basal pelleted diet (the basal experiment diet did not contain chili straw, and the ratio of concentrate to forage was 45:55), and the experimental groups were fed a comprehensive mixed pelleted diet (diets were prepared using a pellet diet preparation machine after the ingredients were crushed and evenly mixed using a blender) including 5%, 10%, 15%, and 20% dried CS with equal energy and nitrogen. The experimental period was 63 days. During the initial seven days, the test animals underwent a dietary adaptation process, and over the next 56 days, normal feeding trials were performed. The basal diet was formulated according to the nutrient requirements of sheep recommended by the NRC (2007), and its composition and nutrient levels are shown in Table 2. During the experiment, the animals were housed and managed under the same conditions, each group of 10 sheep was kept in the same enclosure. They were routinely dewormed, and the breeding environment was disinfected before the experiment. The sheep were fed at 08:00 and 19:30 daily and had free access to food and water. The breeding environment was kept dry and well ventilated. The daily diet intake was adjusted based on the previous day's feed intake to maintain 2%−4% residual feed.

**Table 2:** Composition and nutrient levels of the basal diet (DM basis, %).

**Items**	**Control group**	**CS5% group**	**CS10% group**	**CS15% group**	**CS20% group**
**Ingredients**					
Alfalfa hay	10.88	9.21	7.54	6.38	5.22
Soybean stalk	14.03	12.27	10.45	8.55	6.80
Straw	18.12	16.38	14.49	12.39	10.22
Chili stalk	0.00	5.01	10.00	15.00	20.00
Corn	31.04	30.76	30.75	30.69	30.48
Wheat bran	7.89	7.89	7.74	7.60	7.67
Cottonseed meal	8.88	9.73	10.29	10.71	11.35
Sunflower meal	4.98	4.57	4.57	4.50	4.09
Limestone	0.12	0.13	0.12	0.13	0.11
NaCl	0.53	0.53	0.53	0.53	0.53
CaHPO_4_	0.51	0.50	0.50	0.50	0.51
NaHCO_3_	0.51	0.51	0.51	0.51	0.51
Premix^a^	2.51	2.51	2.51	2.51	2.51
Total	100.00	100.00	100.00	100.00	100.00
**Nutritional level** ^b^					
GE/(MJ/kg)	15.80	16.19	15.69	16.02	15.57
CP	13.11	13.63	13.46	13.74	13.86
EE	2.12	2.56	1.97	2.49	2.45
NDF	55.50	56.65	54.06	56.59	54.81
ADF	25.15	25.37	24.56	26.78	28.52
Ca	1.35	1.57	1.79	1.72	1.63
P	0.47	0.42	0.31	0.48	0.42

^a^The premix provided the following per kg of the diet: Vit A 150,000 IU, Vit D3 56,500 IU, Vit E 8,000 IU, Se (as sodium selenite) 14 mg, I (as potassium iodide) 58 mg, Cu (as copper sulfate) 290 mg, Mn (as manganese sulfate) 1,925 mg, Zn (as zinc oxide) 2,050 mg, and Co (as cobalt sulfate) 24 mg.

^b^Nutritional levels were measured values.

### 2.3 Sample collection

The day after the end of the experiment, the sheep were fasted for 12 h and deprived of water for 2 h, and then five sheep were selected from each group and stunned by electric shock according to the animal welfare protocol, followed by humane euthanasia by bleeding. Rumen fluid was collected from the upper end of the rumen (a small incision was made at the upper end of the rumen using a sterile knife, and the rumen contents were collected. The initial 50 mL of rumen fluid was discarded to prevent contamination, and the remainder was filtered through four layers of gauze into a 50mL centrifuge tube and stored in liquid nitrogen.), and the longest back muscle was collected from the 12th−13th ribs and stored in liquid nitrogen.

### 2.4 Indicator measurement

#### 2.4.1 Slaughter performance

Each experimental sheep was weighed, and the live weight (kg) was recorded before slaughter. The test sheep were bled through the carotid artery and jugular vein, and the head, hoof, skin, and viscera of the test sheep were removed, while the kidneys were retained. The carcass weight was measured and recorded immediately after slaughter. The slaughter percentage was the ratio of the carcass weight to the live weight before slaughter. A longitudinal incision was made on the longissimus dorsi (LD) muscle between the 12th and 13th ribs to obtain the area of the eye muscle. A vernier caliper was used to measure the length of the longest and widest parts of the ocular muscles. The eye muscle area was the product of the length of the eye muscle and width of the eye muscle multiplied by 0.7.

#### 2.4.2 Samples of rumen fluid

The rumen contents were collected and the initial 50 mL of rumen fluid was discarded to prevent contamination. The remainder was filtered through four layers of gauze, and then 30 mL samples of rumen fluid were removed and the pH was measured promptly with a portable pH meter (PHS-3E; Shanghai YiDian Technology Scientific Instrument Co., Ltd., Shanghai, CHN). 10 mL of rumen fluid were transferred into a cryo-storage tube, quickly frozen in liquid nitrogen, and stored for subsequent analysis.

The frozen rumen fluid samples were thawed and centrifuged at 3,000 × g at 4°C for 10 min to obtain supernatants, which were then used to determine the concentrations of ammonia nitrogen (NH_3_-N) (Broderick and Kang, 1980) and volatile fatty acids (VFA) (Erwin et al., 1961), including acetate, propionate, butyrate, isobutyrate, valerate, and isovalerate.

#### 2.4.3 Meat quality

Meat quality measurement included conventional physical indicators (pH, meat color, water loss rate, and cooking loss) and chemical indicators (water, protein, fat, and ash content).

Muscle pH was measured using a portable muscle tissue pH meter (PHS-3E; Shanghai YiDian Scientific Instrument Co., Ltd., Shanghai, CHN), calibrated using standard buffer solutions at pH 4, 6.86, and 9.18. Meat color indices, and the determination was made 45 min after slaughter. L^*^ (brightness), a^*^ (redness), and b^*^ (yellowness), were measured in the dark using a carcase meat color meter (DS-700E; Hangzhou Caipusen Technology Co., Ltd., Zhejiang, CHN). A 1-cm-thick sample was cut vertically along the longest muscle fiber of the back using a circular sampling tool with a diameter of 3 cm (DL-100; Shandong Shunsheng Zhongshi, Shandong, CHN).To measure the loss in weight from cooking, a 3 × 3 × 5 cm piece of meat was dried with a paper towel, weighed (W1, g), placed into a bag, sealed, and heated in a water bath at 85°C for 40 min. After cooling to room temperature, it was patted dry with a paper towel and then weighed (W2, g). Cooking loss was calculated by subtracting W2 from W1 (Ren et al., 2023).

The AOAC method was used to quantitate the moisture content (Method 950.46), the crude protein (Method 928.08), the crude fat (Method 960.39), and the ash (Method 920.153) in the meat (Latimer, 2023).

#### 2.4.4 Fatty acids

Total fatty acids (TFA) in the frozen meat samples were extracted according to the method described by Liang et al. (2023). The fatty acids (FA) were separated by gas chromatography (GC-450, Varian Co., Walnut Creek, CA, USA), and the sample peaks were identified by retention time. The concentrations of individual FAs were determined using a standard curve generated from known standard compounds (mixture of C4–C24 methyl esters; Sigma-Aldrich, Inc., St. Louis, MO, USA).

#### 2.4.5 Amino acids

For determination of amino acid (AA) composition and concentration, 100 mg samples of lyophilized tissues were homogenized in 1.2 mL of 10% sulfosalicylic acid, then centrifuged at 13,500 × g for 15 min at 4°C. Supernatants were removed and filtered through a 0.22 μm filter membrane into a 2.0 mL glass vial. Amino acid composition and concentration in the tissue samples were determined with a highspeed amino acid analyzer (L-8900, Hitachi High-Tech Corporation, TKY, Japan).

#### 2.4.6 DNA extraction, PCR amplification, rumen bacterial sequencing analysis

We utilized the TGuide S96 DNA Kit (Tiangen Biotech Co., Ltd., Beijing, CHN) for DNA extraction from rumen fluid, and amplified the V3-V4 region of the 16S rRNA gene employing a universal primer set comprised of 338F: 5′-ACTCCTACGGGAGGCAGCA-3′ and 806R: 5′-GGACTACHVGGGTWTCTAAT-3′, ensuring that both forward and reverse primers contained a unique Illumina index sequence corresponding to each sample (Duan et al., 2024). We used the method reported by Niu et al. (2023) to configure the PCR reaction mixture with a total of 20 μL. We used the method reported by Li et al. (2023) to detect and analyze the 16S rRNA gene.

### 2.5 Statistical analysis

Excel 2013 was used for preliminary sorting of test data. Statistical analysis was carried out using one-way ANOVA in SPSS 26.0 statistical software (version 26.0; IBM, Armonk, NYC, USA). Multiple comparisons were carried out using Duncan's method. The test results were expressed as the mean, and the degree of variation in each group was expressed as the standard error of the mean (SEM). *P* < 0.05 was considered to be statistically significant. Using Silva.138 as the reference database, we annotated the feature sequences using the naive Bayes classifier to obtain the species classification information corresponding to each feature, and QIIME software was used to generate species abundance at different taxonomic levels. QIIME software was used for bacterial Beta diversity analysis, and Bray-curtis was selected for all distance algorithms. Alpha index plots and species composition plots were drawn with GraphPad Prism (version 10.0, GraphPad Software, Boston, USA) software and Origin (version 2021, OriginLab, Hampton, USA) software, respectively.

## 3 Results

### 3.1 Rumen fermentation

Table 3 shows the effect of CS on rumen fermentation in sheep. There was no significant difference in rumen pH and NH_3_-N concentration between the CON group and the CS groups (*P* > 0.05), but the NH_3_-N concentration showed a linear and quadratic increase trend with the increase of dietary CS supplemental level (0.05 < *P* < 0.10). The concentrations of acetic acid, propionic acid, butyric acid, valeric acid and total acid in the CS10% and CS15% groups showed an increasing trend, but the difference was not statistically significant (0.05 < *P* < 0.10), and the concentrations of acetic acid, propionic acid and total acid showed a quadratic increase with the increase of dietary CS supplemental level (*P* < 0.01 and *P* < 0.05).

**Table 3:** Effect of chili straw on rumen fermentation in sheep (*n* = 5).

**Items**	**Control group**	**CS5% group**	**CS10% group**	**CS15% group**	**CS20% group**	**SEM**	***P*** **-value**		
							**Trt**	**L**	**Q**
pH	6.72	6.70	6.67	6.78	6.57	0.041	0.920	0.542	0.689
NH_3_-N (mg/dL)	14.07	13.49	14.90	14.00	16.94	0.480	0.164	0.064	0.224
Acetate (mmol/L)	59.11	55.81	67.43	63.27	52.79	1.883	0.090	0.671	0.047
Propionate (mmol/L)	15.92	17.82	21.10	18.47	16.65	0.614	0.058	0.586	0.008
Butyrate (mmol/L)	14.10	11.23	16.01	15.04	11.56	0.644	0.053	0.754	0.155
Valerate (mmol/L)	1.57	1.45	1.68	1.83	1.58	0.044	0.076	0.183	0.331
TVFA (mmol/L)	92.48	88.22	106.61	100.99	84.50	2.768	0.052	0.854	0.027
Acetate/propionate	3.79	3.13	3.20	3.49	3.22	0.105	0.257	0.292	0.252

CS5%, 5% chili straw group; CS10%, 10% chili straw group; CS15%, 15% chili straw group; CS20%, 20% chili straw group; SEM, standard error of the mean; NH_3_-N, ammonia nitrogen; TVFA, total volatile fatty acids; Trt, treatment effect; L, linear; Q, quadratic.

### 3.2 Effects of chili straw on abundance, diversity, and composition of rumen bacteria

#### 3.2.1 Venn diagram analysis of rumen bacteria

**Figure 3:**
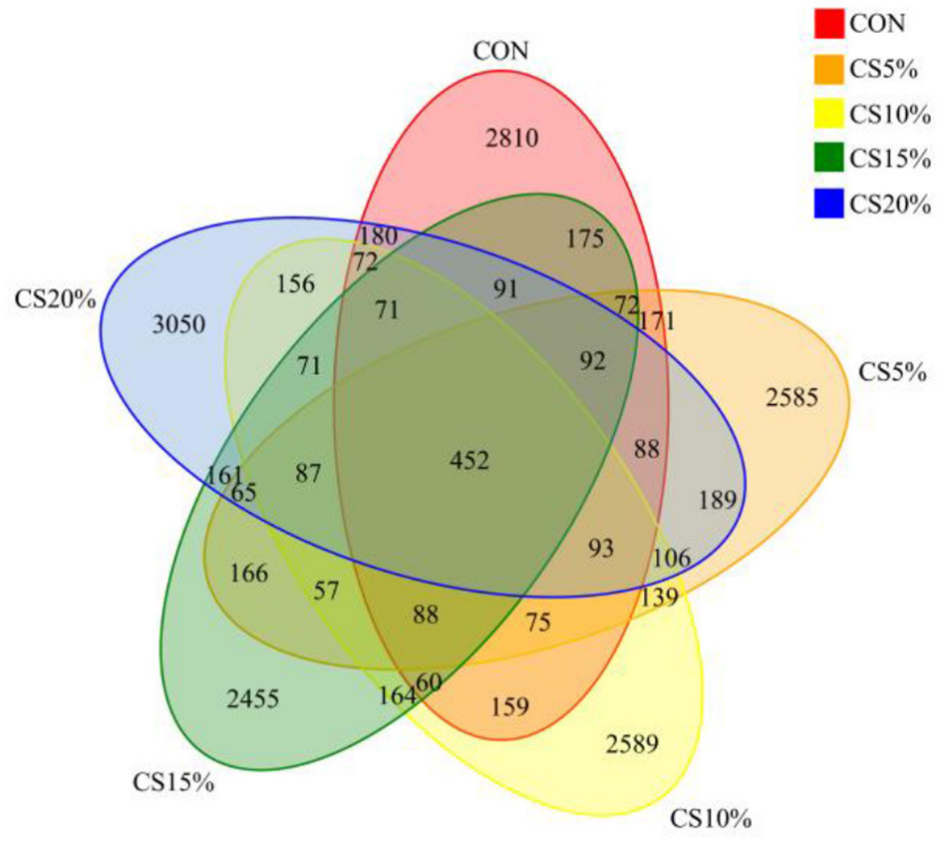
Venn diagram of rumen bacteria.

We sequenced each sample group, and the coverage rates for the resulting data all exceeding 99%, ensuring that the samples collected accurately reflected the changes in the rumen bacterial community. A total of 2,046,084 pairs of reads were obtained from the sequencing of 25 samples. Following quality control and assembly of paired reads, 1,938,871 high-quality clean reads were generated. Each sample generated at least 63,061 clean reads, with an average of 77,555 clean reads. The sequences were clustered at 97% similarity, resulting in 16,789 OTUs (operational taxonomic units) in 25 samples (Figure 3). A total of 452 OTUs were shared between samples from different groups, of which, the CON, CS5%, CS10%, CS15%, and CS20% had 2,810, 2,585, 2,589, 2,455, and 3,050 unique OTUs, respectively, with the CS20% group having a greater number of OTUs compared to the other groups.

#### 3.2.2 Alpha diversity analysis of rumen bacteria

The alpha diversity of rumen bacteria is shown in Supplementary Figures S1A–D. There was no statistically significant difference in the ACE, Chao1, Simpson, and Shannon indices among the groups (*P* > 0.05).

#### 3.2.3 Beta diversity analysis of rumen bacteria

**Figure 4:**
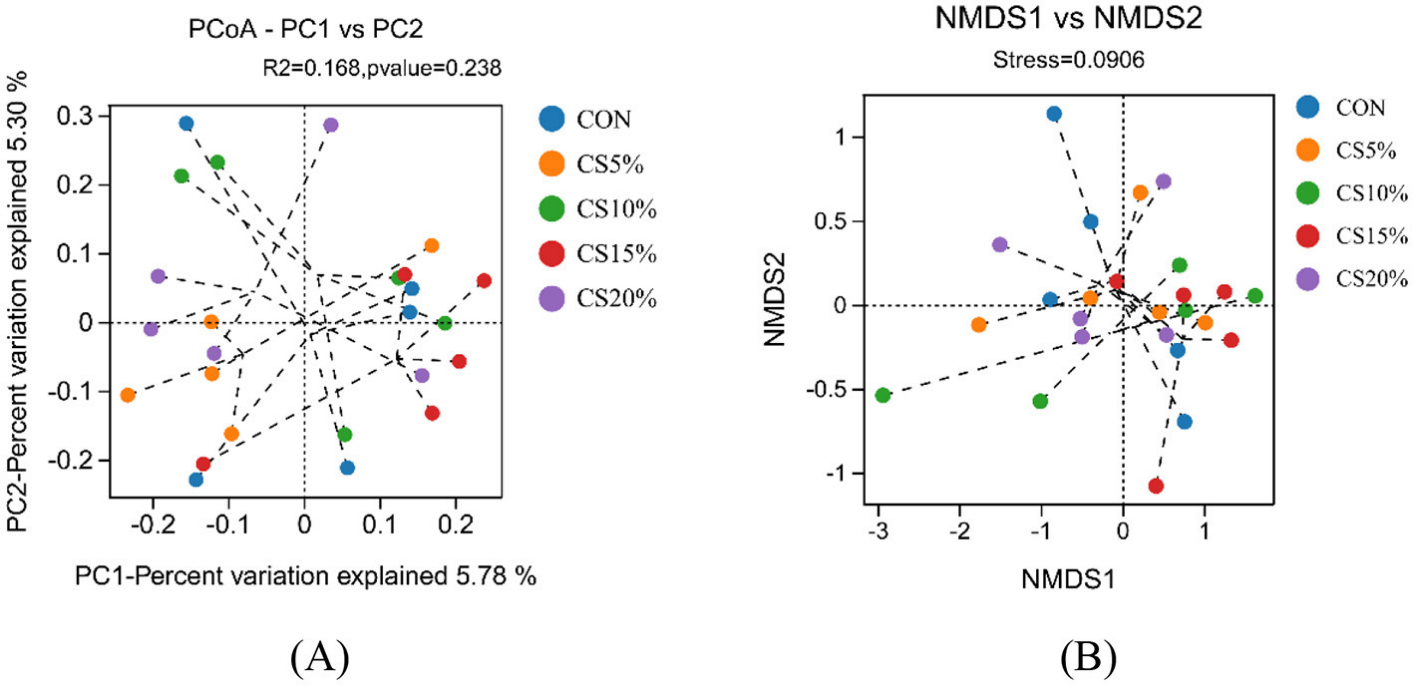
Beta diversity analysis of rumen bacteria (Bray-Curtis distance matrix). **(A)** PCoA principal axis analysis; **(B)** NMDS non-metric multidimensional scaling analysis.

The PCoA analysis of the Bray-Curtis distance matrix (Figure 4A) shows that the points representing rumen microbes in each group were distributed more broadly in the coordinate plane. The addition of CS had no significant effect on the species and abundance of microorganisms in the rumen. The NMDS analysis based on the Bray-Curtis distance matrix (Figure 4B) showed that the CON groups were distantly separated from each other, while the CS15% groups were tightly clustered. The similarity of the rumen bacterial community was higher in the CS15% group than in the CON group.

#### 3.2.4 Analysis of rumen bacterial composition and abundance

**Figure 5:**
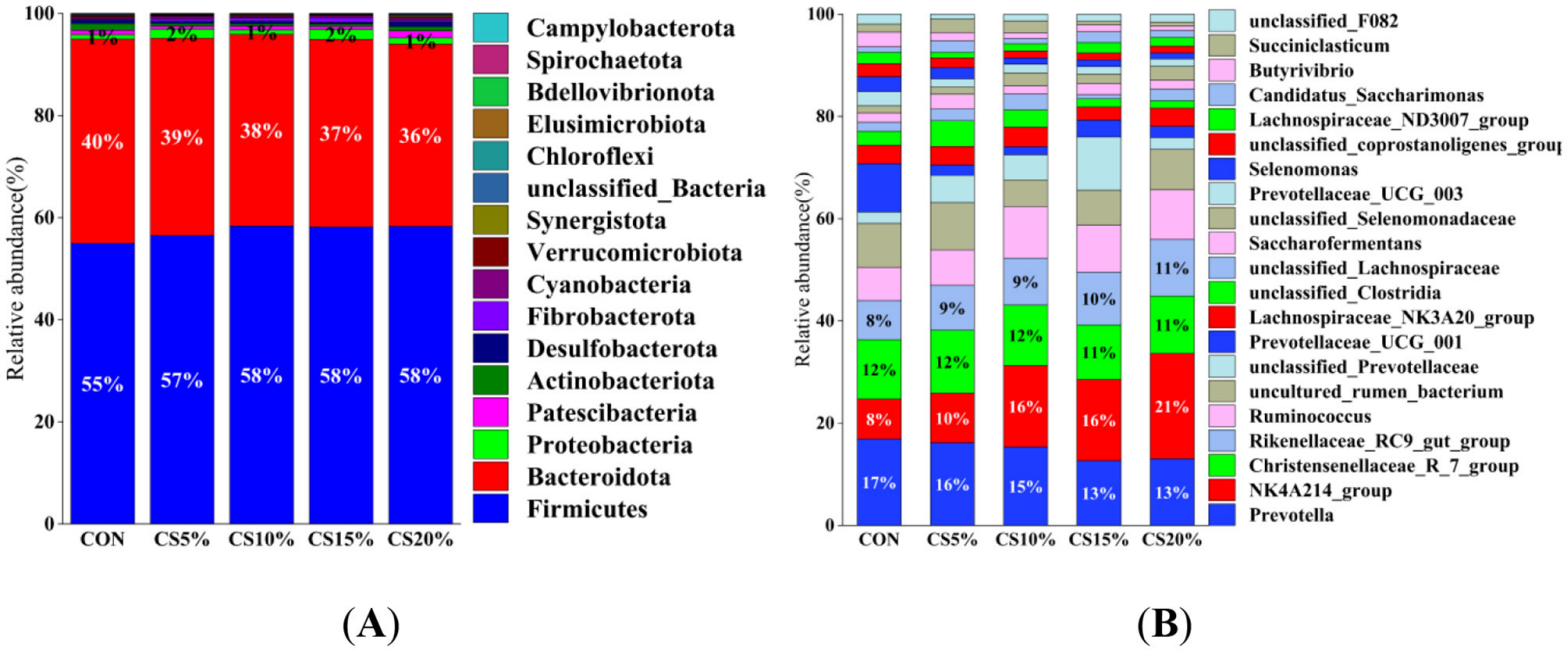
Distribution of bacterial taxa averaged under phyla **(A)** and genera **(B)** levels across the different treatment groups (The Bayesian classifier annotates the feature sequences for classification) (as a percentage of the total sequence).

The structure of the rumen bacterial community is shown in Figures 5A, B. The taxonomic annotation of feature sequences was performed using a plain Bayesian classifier resulting in the identification of 17 bacterial phyla and 26 bacterial from 25 samples of gastrointestinal tract contents from sheep. Firmicutes, Bacteroidota, and Proteobacteria were the dominant phyla, with relative abundances of 57.2%, 38.0%, and 1.4%, respectively, and these three phyla accounted for nearly 97% of the total sequences. Compared with the CON group, the CS group showed trends of increased relative abundance of Firmicutes and Proteobacteria and decreased relative abundance of Bacteroidota, but the differences were not significant (*P* > 0.05). The CS10% group showed an increase in the relative abundance of Firmicutes, while the control group exhibited increased relative abundance of Bacteroidota.

At the genus level, *Prevotella*, the *NK4A214_*group, *Christenellaceae_R_7_*group, and the *Rikenellaceae_RC9_gut_*group were the dominant genera, with relative abundances of 14.8%, 14.2%, 11.6%, and 9.4%, respectively. Compared with the CON group, the relative abundance of *NK4A214_*group and *Rikenellaceae_RC9_gut_*group was increased in the CS group, while the relative abundance of *Prevotella* and *Christensenellaceae_R_7_*group was decreased, but the differences were not statistically significant (*P* > 0.05).

### 3.3 Effect of chili straw feeding on slaughter performance and meat quality

Table 4 shows the effect of dietary addition of CS on the slaughter performance of sheep. The live weight before slaughter (LWBS) of the CS10% and CS15% groups was higher than that of the CS20% group (*P* < 0.05), and the LWBS showed a quadratic increasing with the increase of dietary CS supplemental level (*P* < 0.01). The hot carcass weight (HCW) of the CS10% group was greatly increased compared with that of the CS20% group (*P* < 0.01), and the CS5% group (*P* < 0.05), and the HCW showed a linear and quadratic increasing with the increase of dietary CS supplemental level (*P* < 0.05 and *P* < 0.01). On the other hand, there were no significant differences in the slaughter rate and eye muscle area between the CS group and the CON group (*P* > 0.05).

**Table 4:** Effect of chili straw on slaughter performance of sheep (*n* = 5).

**Items**	**Control group**	**CS5% group**	**CS10% group**	**CS15% group**	**CS20% group**	**SEM**	***P*** **-value**		
							**Trt**	***L***	***Q***
Live weight before slaughter (kg)	44.82^ab^	44.84^ab^	47.48^a^	46.12^a^	41.92^b^	0.557	0.013	0.171	0.004
Hot carcass weight (kg)	22.20^ABab^	21.64^ABbc^	23.54^Aa^	22.34^ABab^	20.06^Bc^	0.319	0.004	0.048	0.004
Slaughter rate (%)	49.53	48.27	49.60	48.41	47.85	0.003	0.092	0.063	0.561
Eye muscle area (cm^2^)	16.43	17.94	17.22	16.62	15.67	0.359	0.354	0.268	0.121

^A, B^Different superscripts indicate significant differences within a row (*P* < 0.01). ^a, b, c^Different superscripts indicate significant differences within a row (*P* < 0.05). SEM is the pooled standard error between five groups; the *P*-value indicates significance.

Trt, treatment effect; L, linear; Q, quadratic.

Table 5 shows the effect of CS on the meat quality of mutton. The cooking loss of the control was larger than that of the CS10%, CS15%, and CS20% groups (*P* < 0.05), and cooking loss in mutton showed a linear and quadratic decreasing with the increase of dietary CS supplemental level (*P* < 0.01 and *P* < 0.05). The redness (a^*^) of the CS15% and CS20% groups was greatly increased compared with the control and the CS5% group (*P* < 0.01) and higher than that of the CS10% group (*P* < 0.05), and the a^*^ showed a linear decreasing with the increase of dietary CS supplemental level (*P* < 0.01). There were no significant differences in L^*^, b^*^, moisture, CP, EE, and ash among the groups (*P* > 0.05), but the b^*^ showed a linear and quadratic decrease trend with the increase of dietary CS supplemental level (0.05 < *P* < 0.10).

**Table 5:** Effect of chili straw on meat quality traits of sheep (*n* = 5).

**Items**	**Control group**	**CS5% group**	**CS10% group**	**CS15% group**	**CS20% group**	**SEM**	***P*** **-value**		
							**Trt**	***L***	***Q***
pH_45min_	6.89	6.96	6.93	6.93	6.96	0.027	0.953	0.637	0.838
Cooking loss (%)	33.79^a^	32.36^ab^	30.09^b^	30.75^b^	31.16^b^	0.398	0.014	0.007	0.024
Luminosity (L^*^)	35.28	35.24	32.27	32.28	33.81	0.785	0.611	0.314	0.375
Redness (a^*^)	17.16^Bb^	17.40^Bb^	17.85^ABb^	18.49^Aa^	18.38^Aa^	0.142	0.002	0.000	0.554
Yellowness (b^*^)	10.00	8.50	8.46	8.33	8.67	0.242	0.157	0.091	0.071
Moisture (%)	74.14	74.01	73.02	74.23	74.04	0.312	0.764	0.993	0.455
Protein (%)	20.78	20.96	21.62	21.38	21.01	0.143	0.355	0.389	0.108
Lipid (%)	3.73	3.83	3.84	3.76	3.81	0.016	0.085	0.389	0.090
Ash (%)	0.84	0.83	0.81	0.82	0.82	0.007	0.767	0.392	0.339

^A, B^Different superscripts indicate significant differences within a row (*P* < 0.01). ^a, b^Different superscripts indicate significant differences within a row (*P* < 0.05). SEM is the pooled standard error between five groups; the *P*-value indicates significance.

Trt, treatment effect; L, linear; Q, quadratic.

### 3.4 Effect of chili straw feeding on fatty acids and amino acids

Table 6 shows the influence of CS on the FAs of mutton. With the increase of dietary CS supplemental level, the content of C14:0 in mutton showed a linear decrease (*P* < 0.05), and the content of C20:1 showed a linear increase (*P* < 0.05), On the other hand, and the content of C16:0 and C18:0 showed a linear decrease trend (0.05 < *P* < 0.10). In comparison with the control, the SFA content in the CS10%, CS15%, and CS20% group decreased (*P* < 0.05), and SFA content in mutton showed a linear decreasing with the increase of dietary CS supplemental level (*P* < 0.05). With the increase of dietary CS supplemental level, the content of PUFA showed a quadratic increase (*P* < 0.05). On the other hand, compared with the control, the PUFA/SFA ratios in the CS group were greatly increased (*P* < 0.01), and the ratio of PUFA/SFA of mutton showed a linear and quadratic increasing with the increase of dietary CS supplemental level (*P* < 0.05 and *P* < 0.01).

**Table 6:** Effect of chili straw on meat fatty acid content in sheep (%) (*n* = 5).

**Fatty acids (% of total fatty acids)**	**Control group**	**CS5% group**	**CS10% group**	**CS15% group**	**CS20% group**	**SEM**	***P*** **-value**		
							**Trt**	***L***	***Q***
C10:0	0.28	0.22	0.23	0.22	0.21	0.017	0.439	0.153	0.323
C12:0	0.20	0.23	0.23	0.23	0.23	0.015	0.985	0.658	0.754
C14:0	3.33	3.12	2.93	2.93	2.92	0.062	0.152	0.024	0.242
C14:1	0.08	0.09	0.10	0.10	0.09	0.004	0.785	0.454	0.314
C15:0	0.48	0.46	0.45	0.45	0.42	0.028	0.842	0.278	0.897
C16:0	26.80	26.68	26.43	26.31	26.29	0.097	0.361	0.051	0.686
C16:1	1.38	1.38	1.56	1.59	1.55	0.048	0.518	0.141	0.574
C17:0	1.40	1.36	1.33	1.35	1.34	0.023	0.895	0.454	0.565
C18:0	24.61	23.85	21.60	21.90	22.52	0.453	0.161	0.055	0.151
C18:1	37.65	36.55	38.73	37.75	37.52	0.361	0.480	0.349	0.646
C18:2	4.08	4.80	4.53	4.53	4.43	0.099	0.226	0.520	0.095
C18:3	0.40	0.46	0.49	0.49	0.47	0.036	0.948	0.539	0.590
C20:0	0.12	0.12	0.11	0.11	0.13	0.004	0.496	0.793	0.194
C20:1	0.06	0.07	0.07	0.07	0.07	0.001	0.105	0.036	0.684
C20:4	0.21	0.22	0.25	0.21	0.24	0.007	0.323	0.257	0.653
SFA	57.22^a^	56.02^ab^	53.30^b^	53.50^b^	54.06^b^	0.515	0.045	0.011	0.102
MUFA	39.17	38.09	40.45	39.50	39.22	0.402	0.505	0.604	0.623
PUFA	4.68	5.49	5.26	5.23	5.14	0.098	0.106	0.305	0.046
PUFA/SFA	0.08^Bb^	0.10^Aa^	0.10^Aa^	0.10^Aa^	0.10^Aa^	0.002	0.003	0.011	0.002

SFA, saturated fatty acid; MUFA, monounsaturated fatty acid; PUFA, polyunsaturated fatty acid.

^A, B^Different superscripts indicate significant differences within a row (*P* < 0.01). ^a, b^Different superscripts indicate significant differences within a row (*P* < 0.05). SEM is the pooled standard error between five groups; the *P*-value indicates significance.

Trt, treatment effect; L, linear; Q, quadratic.

Table 7 shows the effect of chili straw on the AA of mutton. The Gly content in the CS15% group is increased than that in the CON group and CS20% group, respectively (*P* < 0.05), and the Gly content showed a quadratic increasing with the increase of dietary CS supplemental level (*P* < 0.05). The content of EAA, DAA and TAA showed a linear and quadratic increasing with the increase of dietary CS supplemental level (*P* < 0.05), and the content of NEAA SAA showed a quadratic increase trend with the increase of dietary CS supplemental level (0.05 < *P* < 0.10).

**Table 7:** Effect of chili straw on amino acid content in sheep meat (ng/mL) (*n* = 5).

**Amino acids (mg/kg)**	**Control group**	**CS5% group**	**CS10% group**	**CS15% group**	**CS20% group**	**SEM**	***P*** **-value**		
							**Trt**	***L***	***Q***
Gly	58.07^b^	65.50^ab^	66.84^ab^	76.50^a^	61.94^b^	1.940	0.022	0.117	0.016
Ala	253.19	282.58	257.90	264.17	261.67	6.286	0.670	0.970	0.553
Ser	69.62	82.70	92.32	92.25	76.39	3.381	0.091	0.260	0.013
Pro	29.87	38.07	31.90	36.70	31.21	1.305	0.188	0.882	0.128
Val	45.93	56.22	60.19	57.50	52.00	2.361	0.368	0.426	0.065
Thr	36.64	44.19	44.23	47.19	41.44	1.664	0.354	0.291	0.100
Ile	37.35	44.07	50.51	48.39	43.82	1.827	0.185	0.173	0.043
Leu	87.33	101.23	116.82	111.47	99.45	4.089	0.175	0.220	0.035
Orn	15.72	15.57	12.49	14.91	13.78	1.218	0.927	0.629	0.751
Asp	26.07	27.87	28.27	28.77	22.73	1.430	0.701	0.588	0.225
Glu	83.31	90.00	106.61	105.87	96.08	3.709	0.205	0.113	0.104
Lys	68.18	83.31	86.97	90.33	73.03	3.009	0.083	0.393	0.009
Met	34.53	40.93	47.31	43.52	40.33	1.596	0.131	0.19	0.028
His	110.07	133.16	158.35	122.80	129.84	7.515	0.366	0.585	0.151
Phe	47.71	54.97	64.90	60.80	55.84	2.232	0.140	0.148	0.038
Arg	63.46	82.06	76.62	82.20	67.10	2.704	0.070	0.667	0.011
Tyr	45.11	49.41	59.86	60.88	51.81	2.097	0.060	0.072	0.030
Trp	11.03	12.56	12.81	13.01	11.79	0.417	0.574	0.520	0.134
TAA	1,441.04	1,628.29	1,668.25	1,698.21	1,564.91	34.797	0.132	0.178	0.025
EAA	368.70	437.48	483.73	472.20	417.69	16.413	0.174	0.239	0.029
NEAA	1,072.34	1,190.82	1,184.52	1,226.00	1,147.22	21.266	0.193	0.209	0.053
DAA	109.38	117.88	134.88	134.65	118.81	3.906	0.148	0.182	0.043
SAA	458.42	525.59	506.00	531.82	484.43	12.847	0.368	0.524	0.099

TAA, Total animo acid; EAA, essential amino acids; NEAA, non-essential amino acid; SAA, sweet amino acid.

^a, b^Different superscripts indicate significant differences within a row (*P* < 0.05). SEM is the pooled standard error between five groups; the *P*-value indicates significance.

Trt, treatment effect; L, linear; Q, quadratic.

### 3.5 Relationship between ruminal fermentation, meat quality, and rumen bacteria

**Figure 6:**
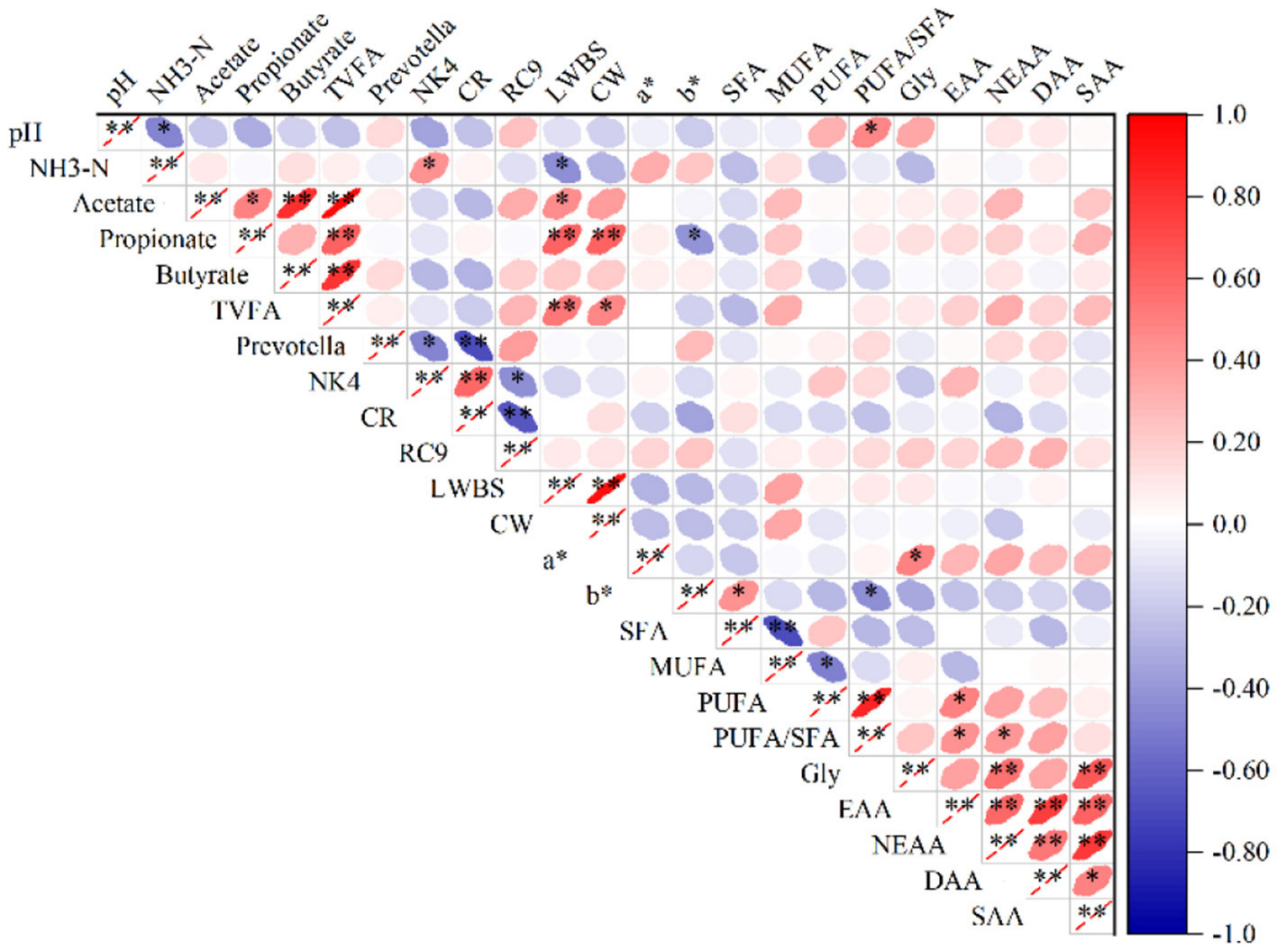
Heatmap showing the relationships among relative microbial abundance, rumen fermentation parameters, and meat quality. *P < 0.05, **P < 0.01. NK4, NK4A214_group; CR, Christensenellaceae_R_7_group; RC9, Rikenellaceae_RC9_gut_group; LWBS, Live Weight Before Slaughter; CW, Hot Carcass weight.

Pearson algorithm was used to calculate the correlation coefficient and build the correlation heat map to illustrate the relationship between rumen flora, rumen fermentation parameters and meat quality. The NH_3_-N level in the rumen had a positive correlation with the relative abundance of the *NK4A214_*group and a negative correlation with LWBS (*P* < 0.05). The rumen pH had a positive correlation with the PUFA/SFA ratio in meat and a negative correlation with rumen NH_3_-N (*P* < 0.05). The acetate content had a positive correlation with the propionate, butyrate, and TVFA contents in the rumen and LWBS (*P* < 0.05 or *P* < 0.01). The propionate level showed a positive correlation with the TVFA content in the rumen, LWBS and CW before slaughter (*P* < 0.01), and b^*^ exhibited a negative correlation (*P* < 0.05). Butyrate showed a positive correlation with the TVFA content in the rumen (*P* < 0.01). The TVFA content in the rumen had a positive correlation with the LWBS and CW (*P* < 0.05 or *P* < 0.01). The relative abundance of *Prevotella* was negatively correlated with the relative abundance of the *NK4A214_*group and the *Christensenellaceae_R_7_*group in the rumen (*P* < 0.05 or *P* < 0.01). The relative abundance of the *NK4A214_*group showed a positive correlation with the *Christensenellaceae_R_7_*group and a negative correlation with the *Rikenellaceae_RC9_gut_*group in the rumen (*P* < 0.05 or *P* < 0.01). The relative abundance of the *Christensenellaceae_R_7_*group was negatively correlated with the *Rikenellaceae_RC9_gut_*group in the rumen (*P* < 0.01). The LWBS was positively correlated with the CW. The a^*^ exhibited a positive correlation with the Gly content in the meat (*P* < 0.01). The b^*^ had a positive correlation with the SFA content, and a negative correlation with the PUFA/SFA ratio in the meat (*P* < 0.05). The SFA showed a negative correlation with the MUFA content in the meat (*P* < 0.01). The MUFA had a negative correlation with the PUFA content in the meat (*P* < 0.05). The PUFA content revealed a positive correlation with the PUFA/SFA ratio and EAA content (*P* < 0.05 or *P* < 0.01). The PUFA/SFA was positively correlated with the EAA and NEAA in the meat (*P* < 0.05). Gly revealed a positive correlation with the NEAA and SAA contents (*P* < 0.01). The EAA had a positive correlation with the concentrations of NEAA, DAA and SAA in the meat (*P* < 0.01). The NEAA showed a positive correlation with the DAA and SAA contents (*P* < 0.01), and the DAA had a positive correlation with the SAA concentration (*P* < 0.05) (Figure 6).

## 4 Discussion

### 4.1 Influence of chili straw feeding on rumen fermentation of sheep

The rumen pH reflects the fermentation activity and physical state of the rumen and is considered to be a key determinant of rumen homeostasis (Ran et al., 2024). The pH value is affected by multiple factors including feed type, salivary secretion, and microbial types (Jin et al., 2016). The normal pH in the rumen is between 6.0 and 7.0. Previous studies have shown that air-dried CS can increase the pH in sheep rumen (Cheng Z. Z. et al., 2023). Here, the rumen pH of each group was within the normal range of 6.5–7.0, which indicated that CS had no adverse effects on rumen fermentation. This is consistent with published research results.

The rumen NH_3_-N is the main nitrogen source for microbial growth and microbial protein synthesis (Cui et al., 2023). It reflects the efficiency of microorganisms in degrading nitrogen-containing substances and utilizing ammonia, and is also an important indicator of balanced nitrogen metabolism (Preston and Leng, 1988). It was reported that the rumen NH_3_-N level suitable for bacterial growth was 5–30 mg/100 mL (Lv et al., 2020). The NH_3_-N concentration in the CS group was slightly higher than in the control, which was in agreement with previous studies. The CS10% group showed a significant increase in the apparent nutrient digestibility of CP by sheep and improved nitrogen utilization (Li et al., 2024). The cellulose-degrading microbes in the rumen secrete a large amount of cellulase, which promotes the decomposition of cellulose in the straw and provides sufficient substrate for the growth and reproduction of microorganisms.

More than 70% of the energy supply for ruminants is provided by VFA, which mainly come from carbohydrates in the feed (Anantasook et al., 2013). During rumen fermentation, propionate provides energy for the body, while acetate and butyrate are converted into methane and carbon dioxide, reducing energy efficiency (Lam et al., 2017; Zhou et al., 2018). On the other hand, propionate and butyrate compete with methane for hydrogen during fermentation, reducing methane energy consumption, and improving energy supply (Gunun et al., 2017). Acetate, butyrate, and propionate are derived from different fermentation substrates, the first two mainly through the fermentation of cellulose, while the last is through the fermentation of sugars and starch (Pragna et al., 2018). In this study, the concentrations of acetic acid, propionic acid and TVFA in the rumen of the CS group were higher than those of the control, among which the content of acetic acid, propionic, butyric acid, and TVFA in the CS10% group were the highest, indicating that the CS10% group could not only effectively promote the cellulose-degrading bacteria in the rumen to secrete a large amount of cellulase to degrade cellulose in the straw, but also increase the concentration of propionate, thereby supplementing the energy supply (Lv et al., 2020).

### 4.2 Effects of chili straw on rumen bacteria of sheep

The rumen microbes are principally involved in energy generation in ruminants. Research has shown that changes in rumen microbial communities can modulate energy efficiency (Astawa et al., 2011; Dórea et al., 2020). In this study, the alpha diversity index did not differ significantly between the control and CS groups. Firmicutes and Bacteroidota are the predominant ruminal microorganisms. The combined populations of the two phyla account for more than 70% of rumen microorganisms and are considered to be important microorganisms that provide energy to ruminants (Shi et al., 2014; Xue et al., 2020). We found that the Firmicutes and Bacteroidota phyla showed the highest relative abundance, and the together accounted for >70% of rumen bacteria also in the present study. Bacteroidota is an important bacterial group in the rumen, which can promote the breakdown of polysaccharide, protein, and carbohydrate in fodder, mainly converting non-fibrous substances into acetic acid, propionic acid and other substances (Tremaroli and Bäckhed, 2012; Liu et al., 2016). Firmicutes are involved in energy generation by synthesizing an array of enzymes to digest nutrients. They are a key bacterial family for improving fiber utilization (Söllinger et al., 2018). The high NDF content in the CS groups stimulated the growth of Firmicutes.

The dominant genera included *Prevotella*, the *Christensenellaceae_R_7_*group, the *NK4A214_*group, and the *Rikenellaceae_RC9_gut_*group. The members of the *Christensenellaceae_R_7_*group are *Firmicutes* and are primarily associated with the breakdown of cellulose and hemicellulose (Petri et al., 2019). The research of Ran et al. (2021) provided evidence that the relative abundance of the *Christensenellaceae_R_7_*group in the rumen of yaks increased when the concentrate-to-roughage ratio was 50:50 compared with 80 to 20. The relative abundance of the *Christensenellaceae_R_7_*group in our study disagreed with the above findings. With the increase in CS, the abundance of the *Christensenellaceae_R_7_*group in sheep rumen showed a decreasing trend. The appropriate cellulose content can stimulate the proliferation of the *Christensenellaceae_R_7_*group, while excessive cellulose did not increase proliferation of the *Christensenellaceae_R_7_*group because of the lack of corresponding enzymes for substrate secretion. *Prevotella* is the dominant genus in the rumen (Bowen et al., 2018), and it is principally involved in digestion of cellulose, starches, hemicellulose and proteins (Li and Guan, 2017), and shows a positive correlation with acetate (Zhou et al., 2022). The *Rikenellaceae_RC9_gut_*group belongs to the *Rikenellaceae* family and is the main rumen microorganism. It secretes a large amount of cellulase and hemicellulase to degrade cellulose and hemicellulose in the feed (Zhu et al., 2020). It is essential for fiber digestion, and its relative abundance corresponds to the fiber content of the feed (Wang et al., 2019). Studies have shown that when the neutral detergent fiber content in the diet is decreased, the relative abundance of the *Rikenellaceae_RC9_gut_*group in the rumen also drops (Zened et al., 2012). Therefore, the high content of NDF in CS is likely the reason for the high relative numbers of the *Rikenellaceae_RC9_gut_*group in the rumen. The *NK4A214_*group is a member of the *Ruminococcaceae* family and is related to the degradation of fiber substances. It can produce cellulase that breaks down crude fiber, and improves the digestion of high-fiber feed by ruminants. Studies have shown that the *NK4A214_*group is the main genus of the microbiota and improves the digestibility of ADF (Niu et al., 2022). Here, the relative population of the *NK4A214_*group in the CS-fed animals was higher than control, which agreed with previous findings (Li et al., 2024).

### 4.3 Effects of chili straw on slaughter performance and meat quality of sheep

The slaughter performance reflects the digestibility of the feed and the growth capacity of the animal, and is usually measured as live weight before slaughter, hot carcase weight, slaughter rate and eye muscle area. In our study, the pre-slaughter live weight of the CS10% and CS15% groups was significantly higher than that of the control, and the hot carcase weight of the CS10% group was extremely significantly higher than that of the CS20% group. The changes in pre-slaughter live weight and hot carcase weight are most likely related to the bioactive components of chili straw. This may be because chili straw is rich in flavonoids, organic acids and polysaccharides, which can improve protein content of the feed, pro-mote the metabolism and utilization of nutrients, and thus improve the slaughter performance of sheep (Su et al., 2022). However, the very high fiber content of concentrated chili straw (CS20%) will have a certain impact on digestion and metabolism and could have an adverse effect on the slaughter performance of sheep, by reducing carcase weight and slaughter rate. In addition, carcase weight is also related to the concentrations of acetate, propionate, and total volatile fatty acids in the rumen. High concentrations of propionate and low acetate/propionate ratios indicate higher energy utilization (Poudel et al., 2019). This is consistent with the above results that propionic acid and TVFA concentrations were positively correlated with live weight and hot carcase weight before slaughter.

The pH directly affects the water holding capacity and color of meat, and reflects the rate and magnitude of muscle glycolysis after slaughtering (Su et al., 2022). The higher the pH, the slower the rate of muscle glycogen breakdown, the less water exuded, the more stable the protein structure, and the better the preservation of the meat (Su et al., 2022). The water loss rate depends on the water holding capacity of muscles. Early research showed that adding flavonoid-rich Allium mongolicum Regel to the diet of small-tailed Han sheep significantly increased the pH over 24 h and reduced cooking losses (Liu et al., 2019). Here, the pH of the CS group exhibited an upward trend at 45 min, and the water loss rate and cooking loss were significantly reduced, indicating that the CS content improved mutton quality. CS is rich in antioxidants and bioactive substances like polysaccharides and flavonoids that can enhance the stability of cell membranes, reduce calcium content, increase pH, allow Ca2^+^ to penetrate into muscles, reduce the rate of glycolysis, retard the oxidative degeneration of myofibril proteins, and reduce the loss of water (Baldi et al., 2018). Meat color is a direct reflection of muscle physiology and biology and is determined by myoglobin (Mb) and hemoglobin, which are affected by oxidation. What affects the a^*^ value are Mb and the oxidation product of oxygenated Mb, metmyoglobin (MetMb). The b^*^ parameter is influenced by the amount of carotenoids in the diet and the fat content between muscles (Nieto et al., 2009). Research has proven that adding 800 mg/kg of apple polyphenols to the diet of finishing pigs can reduce the L^*^ and b^*^ values of the longissimus thoracicus muscle (Xu et al., 2019). Our results show that the a^*^ values of the CS15% and CS20% groups were significantly higher than those of the CS10% group. The L^*^ and b^*^ values showed a decreasing trend, which is consistent with Hughes et al. (2019). Adding wheat straw to Tibetan sheep feed increases the a^*^ value and reduces L^*^ and b^*^. It shows that the bioactive components in CS could reduce Mb oxidation by competing with Lipopolysaccharide (LPS) for oxidized Mb in muscles. The phenolic hydroxyl groups and carbonyl groups in flavonoids can react with metal ions to block free radicals, thereby increasing the activity of MetMb reductase and delaying the oxidation of Mb (Chen et al., 2020). Therefore, the flavonoid compounds in CS can improve the color of meat and increase its marketability.

### 4.4 Effects of chili straw on fatty acids and amino acids in lamb

FAs in lamb meat are cruciain determining meat flavor (Ma et al., 2015). Studies have shown that C18:0 can enhance the mutton odor, while flavonoids can reduce it by decreasing the activity of enzymes related to fatty acid (C18:0) metabolism (Liu and Ao, 2020). In the present study, CS decreased C16:0 and C18:0 and increased C18:1, C18:2, C18:3, and C20:4 in lamb. The flavonoids present in CS may have contributed to this effect. The flavor of meat is also affected by unsaturated fatty acids (UFAs) in muscle, and UFAs are more likely to produce unsaturated aldehydes when heated, which improves the flavor (Su et al., 2022). In this study, CS increased the concentration of UFAs, MUFAs, and PUFAs in muscle and decreased SFAs. Previous studies have shown that bioactive compounds in alfalfa-based feed can increase the activity of enzymes involved in SFA synthesis and promote the conversion of SFA to UFA (Kwiecień et al., 2020). This may explain how flavonoids promote the increase of UFA and the decrease of SFA in sheep muscle.

The FA content in feed is related with human physiological metabolism, as it determines the nutrient values in meat products and improves the quality and edibility of meat products. The fatty acid composition and polyphenol content of feed can affect the fatty acid profile of meat (Maryna et al., 2016). MUFAs have been shown to protect the heart, lower blood sugar, and regulate blood lipids, while PUFAs are active in lowering blood lipids, inhibiting platelet aggregation, resisting autoimmune reactions, and promoting body development. We found that CS increased the concentration of UFAs, MUFAs, and PUFAs and the ratio of PUFA/SFA in muscle. C16:1 and C18:1 are MUFA; C16:1 efficiently regulates glucose and lipid metabolism and reduces inflammation, while C18:1 reduces the levels which is consistent with the results of Ma et al. (2015) who found that feed supplemented with sea buckthorn flavonoids increased the concentration of C16:1 and C18:1 in muscle. C18:2 and C18:3 are PUFAs, and when biohydrogenated, form the major isomer of c9, t11-conjugated linoleic acid (CLA) in the rumen. Conjugated linoleic acid can improve immunity, exert antitumor and anti-atherosclerotic effects, prevent diabetes, and reduce triglyceride and cholesterol levels in animals and humans. Among the PUFAs, C18:2 has a positive role in lowering blood cholesterol and preventing atherosclerosis, while C18:3 is instrumental in lowering blood lipids and glucose levels, the development of the brain and retina, and as an antioxidant, an antibacterial, and an anti-inflammatory (Joris et al., 2019). In this study, CS feeding resulted in elevated levels of C18:2 and C18:3 in muscle, which is consistent with the results of Arend et al. (2022). Adding dried grape pomace to the diet can increase the C18:2 content in the loin muscle of calves. The above studies show that CS has the potential to improve the FA composition of muscle and enhance the nutritional quality and aroma of lamb, while also increasing its edibility. It is possible that the active ingredients in CS (flavonoids, polysaccharides, and polyphenols) have antioxidant activity and can reduce the hydrogenation of rumen bacteria, resulting in greater deposition of MUFAs and PUFAs in the muscles of sheep (Tan et al., 2010).

The types and contents of amino acids in muscles are important indicators of their nutrient value. The high level of EAAs in mutton supports its good nutritional value. EAAs are required by the human body as they cannot be synthesized and must be obtained through food; thus, foods high in EAAs have good nutrient value. The levels of certain AAs are significantly linked to proper metabolic functioning within the human body. To illustrate this, lysine is the first limiting AA in the human diet and functions in promoting growth, development and physical health. After heat treatment, it produces substances such as hydrogen sulfide and thiophene, which increase the flavor of meat (Ma et al., 2019). Arginine is capable of enhancing the immune response by stimulating the proliferation of white blood cells and macrophages, which in turn boosts overall immunity, and leucine boosts the activity of antioxidant enzymes, thus improving the body's ability to combat oxidative stress (Katayama and Mine, 2007). Ser is involved in lipid metabolism and in the synthesis of Met, Gly and Cys, and also is necessary for proper functioning of the immune and central nervous systems. In this study, CS increased the content of EAAs such as Lys, Trp, and Leu, and NEAAs such as Gly, Ala, Glu, Ser, and Tyr in mutton. This supports our hypothesis that CS can improve the nutrient value of mutton in human nutrition, because it contains abundant digestible protein and an ideal AA ratio.

The AA content in muscle has a crucial impact on the nutrient value of meat, and some functional AAs are also important flavor enhancers. AAs can also participate in Maillard reaction with reducing sugars, which is one of the principal ways of ensuring flavorful meat. The main flavor-generating AAs are Asp and Glu, whose sour taste combines with the salty taste of sodium ions during hydrolysis to produce the meaty umami flavor (Ramalingam et al., 2019). The main sweet amino acids are Ala, Pro, Gly, Ser, and Thr. In this study, the addition of CS increased the concentration of flavor-generating AAs such as Glu, Ser, Asp, Gly, and Ala in the muscle, and enhanced the umami flavor and overall taste of the meat. At present, there are no reports on the effect of CS on the content of the flavor-generating AAs in mutton. This study shows that adding CS to the feed is beneficial as it enhances mutton quality by increasing the concentration of flavor-boosting amino acids in the meat. This may be because CS affects the utilization rate of nitrogenous compounds and the conversion rate of protein in sheep, promoting the deposition of flavorful AAs and improving the aroma of cooked mutton.

## 5 Conclusions

The results of this study supported our hypothesis that adding chili straw to animal feed could improve rumen fermentation and rumen microbial community structure. At the same time, CS can also significantly increase pre-slaughter live weight and carcass weight and improve slaughter performance. Importantly, adding CS to the diet significantly changed the quality of mutton, increasing muscle pH, dressing rate, and reducing cooking loss; it also increased a^*^ values and reduced b^*^ values, thereby improving meat color. In addition, CS can increase the protein and fat content of mutton, improve the con-tent of EAAs and flavorful amino acids, and help increase the content of the MUFAs, C16:1 and C18:1, and the PUFAs, C18:2 and C18:3, thereby enhancing the nutrient value of the mutton. In summary, the recommended dietary supplement amount of CS is 10%. The above results provide an empirical foundation for the use of CS as a safe, effective, and green roughage additive in the mutton industry. At the same time, it provides a scientific basis for the production of high-grade mutton to meet consumers' demands for premium-quality functional livestock products.

## Data Availability

The rumen bacteria data were deposited into the National Center for Biotechnology Information (NCBI) Sequence Read Archive (SRA) with the accession numbers PRJNA1170910, PRJNA1170932, and PRJNA1203874. The other original data were uploaded to Figshare: https://doi.org/10.6084/m9.figshare.28104533.v1.
