# Supplementary material for: Effects of chili straw on rumen fermentation, meat quality, amino acid and fatty acid contents, and rumen bacteria diversity in sheep
Source: Front Microbiol. 2025 Jan 14;15:1525612. doi: 10.3389/fmicb.2024.1525612 (PMC11773153; doi:10.3389/fmicb.2024.1525612)
Supplement: Supplementary file 1 [file Data_Sheet_1.docx]

Supplementary Materials

| 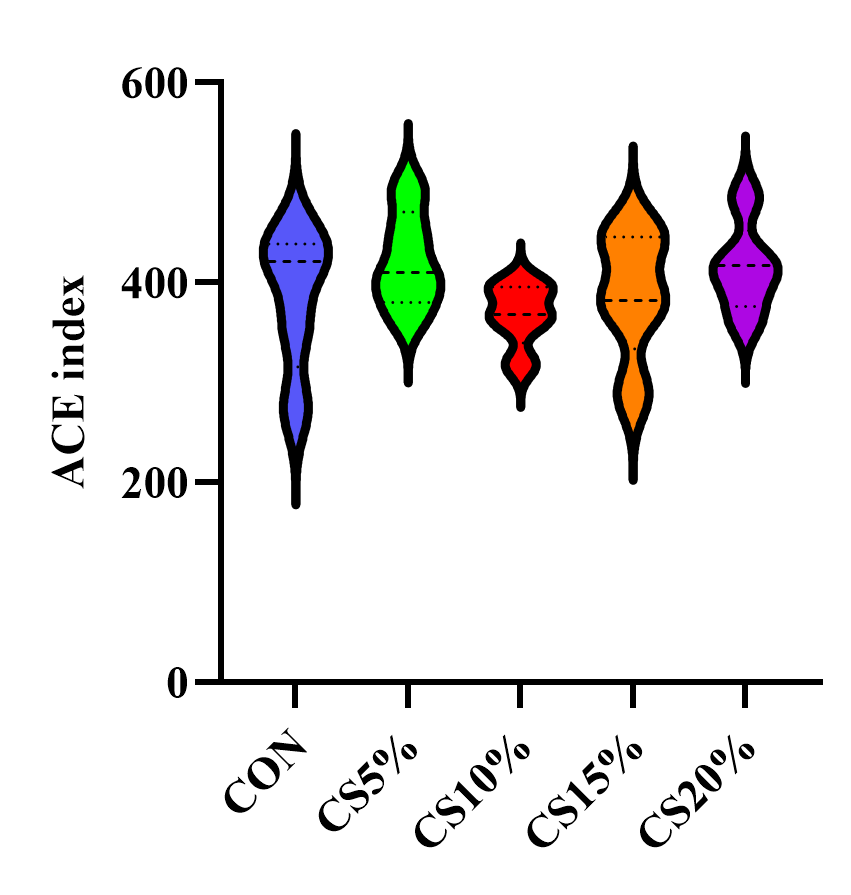 | 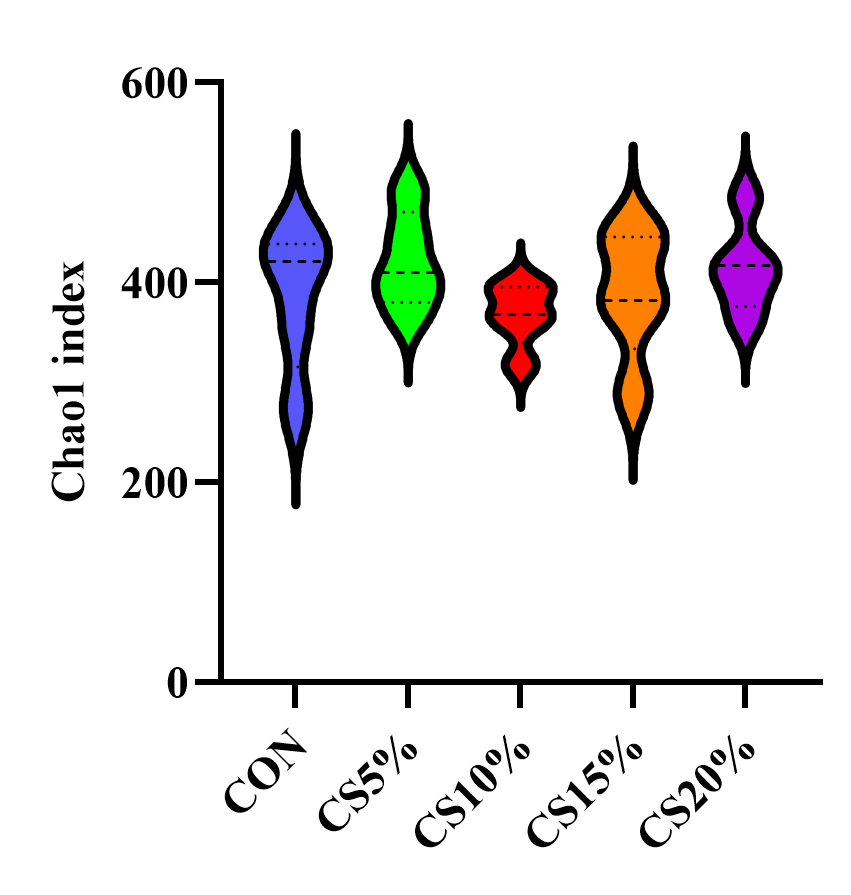 |
| --- | --- |
| (A) | (B) |
| 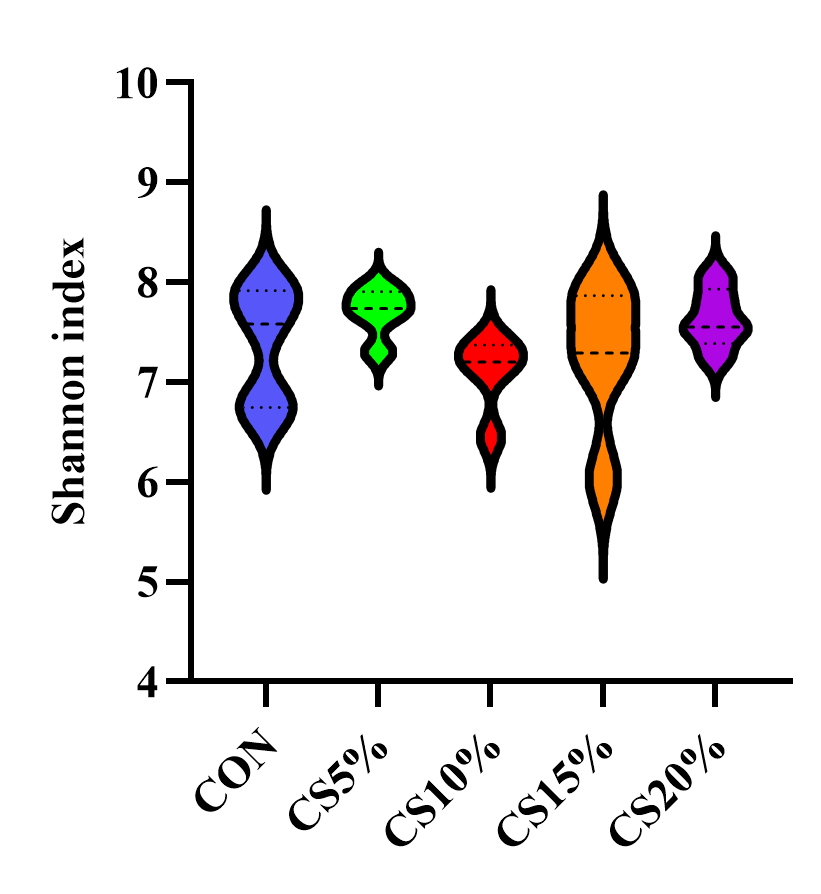 | 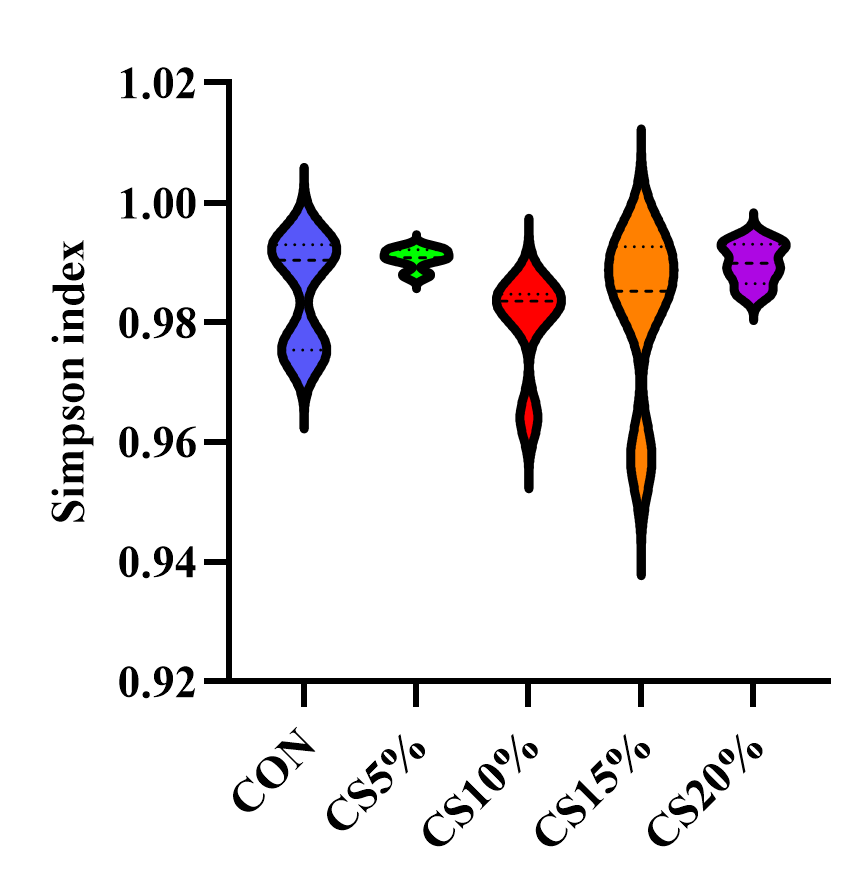 |
| (C) | (D) |

**Supplementary Figure S1.** Alpha diversity analysis of rumen bacteria. (A) ACE index of species richness; (B) Chao1 index of species richness; (C) Shannon index of species diversity; (D) Simpson index of species diversity.
